# Research on the interaction of astragaloside IV and calycosin in *Astragalus membranaceus* with HMGB1

**DOI:** 10.1186/s13020-023-00789-7

**Published:** 2023-07-04

**Authors:** Junyi Ye, Yong Huang, Xuewa Jiang, Pingping Shen, Chaofeng Zhang, Jian Zhang

**Affiliations:** 1 Department of Resources Science of Traditional Chinese Medicines and State Key Laboratory of Natural Medicines, School of Traditional Chinese Pharmacy, China Pharmaceutical University, Nanjing, 210009 People’s Republic of China; 2 Jiangsu Key Laboratory of TCM Evaluation and Translational Research, China Pharmaceutical University, Nanjing, 211198 People’s Republic of China; 3 State Key Laboratory of Natural Medicines, China Pharmaceutical University, 24# St. Tong Jia Xiang, Nanjing, 210009 China

**Keywords:** *Astragalus membranaceus*, Small molecule-protein interaction, High mobility group box 1 (HMGB1), Astragaloside IV, Calycosin

## Abstract

**Background:**

High mobility group box 1 protein (HMGB1), a lethal late inflammatory mediator, contributes to the pathogenesis of diverse inflammatory and infectious diseases. Astragaloside IV and calycosin as active ingredients in *Astragalus membranaceus*, possess potent regulatory ability on HMGB1-induced inflammation, however, the interaction between these two phytochemicals and HMGB1 has not been elucidated yet.

**Methods:**

To further investigate the interaction of astragaloside IV, calycosin with HMGB1 protein, surface plasma resonance (SPR) and a series of spectroscopic methods, including UV spectra, fluorescence spectroscopy, circular dichroism (CD), were used. Molecular docking was also carried out to predict the atomic level’s binding modes between two components and HMGB1.

**Results:**

Astragaloside IV and calycosin were found to be able to bind HMGB1 directly and affect the secondary structure and environment of the chromogenic amino acids of HMGB1 to different extents. *In silico*, astragaloside IV and calycosin showed a synergistic effect by binding to the two independent domains B-box and A-box in HMGB1, respectively, where hydrogen and hydrophobicity bonds were regarded as the crucial forces.

**Conclusion:**

These findings showed that the interaction of astragaloside IV and calycosin with HMGB1 impaired its proinflammatory cytokines function, providing a new perspective for understanding the mechanism of *A. membranaceus* in treating aseptic and infectious diseases.

## Introduction

*Astragalus membranaceus Bunge* (*Leguminosae*) is one the most widely used herbal materials in Traditional Chinese Medicine (TCM) for preventing and treating cognitive impairment, diabetes, viral infection, fatigue, and anorexia [1, 2]. The main chemical constituents of *A. membranaceus* are triterpenes, saponins, and flavonoids with various biological effects, including immunoregulatory, hepatoprotective, antioxidant, anti-hypoxic and other [3]. Astragaloside IV and calycosin glycosides are the major active ingredients contributing to their clinical therapeutic effects and are tentatively regarded as the chemical markers for the quality control of *A. membranaceus* in the Pharmacopoeia of the People’s Republic of China [4]. Astragaloside IV is a cycloartane-type triterpene glycoside with a similar skeleton structure to steroids, usually used as an anti-cancer and anti-diabetic drug due to its potency in inhibiting the expression of interleukin (IL)-1, IL-6 and high mobility group box 1 (HMGB1), as well as the production of nitric oxide (NO) [5]. Calycosin as an *O*-methylated isoflavone and phytoestrogen found abundantly in *A. membranaceus*, displays multiple biological activities, such as anti-oxidative stress, anti-inflammatory, antibacterial, and proangiogenic effects [6]. In addition, calycosin also alleviates sepsis-induced acute lung injury by inhibiting the HMGB1-myeloid differentiation primary response protein 88 (MyD88)-nuclear factor-κB (NF-κB) pathway and NOD-like receptor thermal protein domain associated protein 3 (NLRP3) inflammasome [7].

HMGB1 is a DNA-binding protein localized inside the nucleus, which is released in large quantities during cell injuries or infections [8]. In extracellular space, HMGB1 acts as a damaged associated molecule pattern (DAMP) and a late mediator of lethal endotoxemia and sepsis that contributes to the pathogenesis of inflammatory and infectious diseases by interacting with multiple receptors, including toll-like receptor 4 (TLR4), receptor of advanced glycation and end products (RAGE) [9, 10]. HMGB1-TLR4/RAGE interaction activates NF-κB and mitogen-activated protein kinase (MAPK) signaling pathway, leading to a massive release of pro-inflammatory cytokines, chemokines, and adhesion molecules [11]. Blocking the HMGB1-TLR4/RAGE axis with natural or synthetic small molecule inhibitors, e.g., ethyl pyruvate [12], glycyrrhizin [13], or metformin [14, 15] can reduce the onset of HMGB1-involved in inflammatory cascades. Besides, the phytochemical calycosin and astragaloside IV in *A. membranaceus* have been reported to inhibit HMGB1-related inflammation [16], but the intermolecular interactions between the two phytochemicals and HMGB1 are yet to be explored.

In this study, we first evaluated the inhibitory effect of calycosin and astragaloside IV in the HMGB1-induced inflammatory cell model. It is well known that the curative effect of TCM is considered as the synergic activity of their multiple bioactive compounds [17]. In light of this, the synergy effect between these two ingredients on the pro-inflammatory effect of HMGB1 was studied. To further investigate the inhibition mechanism, the binding affinity of astragaloside IV, calycosin with HMGB1 protein was determined by surface plasma resonance (SPR) technique. Subsequently, a series of spectroscopic methods, including UV spectra, fluorescence spectroscopy, and circular dichroism (CD), were used to explore the characteristics of small molecules with HMGB1 interaction and their effects on the spatial conformation of HMGB1 in the physiological state. Molecular docking was also carried out to predict the atomic level’s binding modes between two components and HMGB1. The present work was aimed to understand the underlying molecular mechanisms of *A. membranaceus* in the treating infection- and injury-related inflammatory diseases.

## Experimental section

### Materials and methods

Astragaloside IV and calycosin were purchased from Nanjing Plant Origin Biological., PR China, with a purity of 98%. HMGB1 was purchased from Sino Biological Inc (Beijing, China), with the purity of HMGB1 over 90% determined by SDS-PAGE. The absorption spectra were detected on Cary Series ultraviolet-visible (UV–vis) spectrophotometer (Agilent Technologies, USA). The excitation and emission spectra were scanned on Cary Eclipse Fluorescence Spectrophotometer. The CD spectrum was measured on JASCO 810 spectropolarimeter. Other experimental chemicals were purchased from Sinopharm Chemical Reagent Co., Ltd (Beijing, China).

### Inhibition on HMGB1-induced NO release

RAW 264.7 cells (1.0 × 10^5^ cells/well) were inoculated in 96-well plates and cultured in an incubator containing 5% CO_2_ at 37℃ for 12 h. Test compounds (100, 50, 25, 12.5, 6.25, 3.125 µM) and HMGB1 (1 µg/mL) were co-incubated in vitro for 1 h at 4 ℃ and then given to the cells as the administration group. For the model group, 100 µL dulbecco’s modified eagle medium (DMEM) medium, including 1 µg/mL HMGB1 was added. Free serum DMEM was used as the blank group. The culture was continued at 37 ℃ in an incubator containing 5% CO2 for 24 h. Take 100 µL culture supernatant of each well into another new 96-well plate, and then add 50 µL Griess reagent A and 50 µL Griess reagent B in turn. The mixture was fully shaken and mixed, and the OD value at 540 nm was detected with a microplate reader (Tecan Trading AG, Switzerland). The NO concentration in the cell culture supernatant can be calculated by NaNO_2_ standard curve. The cell viability was detected using the conventional 3-[4,5]-dimethylthiahiazo (-z-y1)-3,5-di- phenytetrazoliumromide (MTT) assay. Discard the supernatant and add 100 µL MTT (0.5 mg/mL). After incubation at 37 ℃ for 2 h, the reaction was terminated by adding 150 µL Dimethyl sulfoxide (DMSO). The amount of MTT formazan product was recorded by measuring absorbance at 570 nm test wavelength and 630 nm reference wavelength using a micro-plate reader.

### Binding affinity by surface plasmon resonance spectroscopy (SPR)

SPR is a biosensor technology widely used to study small molecule-protein interaction with the advantages of high sensitivity, low consumption, label-free and real-time detection. Herein, the affinity between astragaloside IV, calycosin and HMGB1 was detected on the Biacore T200 instrument. The first and third channels of the CM5 chip were set as a reference channel, and the second and fourth channels as detection channels immobilized with HMGB1. CM5 chip was activated with 1-(3-Dimethylaminopropyl)-3-ethyl carbodiimide hydrochloride (EDC)/N-Hydroxysuccinimide (NHS) solution for 7 min. The HMGB1 protein was diluted to 100 µg/mL with 10 mM sodium acetate solution (pH = 5.0) and was coupled to the second channel with the coupling amount of 4500 response units (RU) and flow rate of 10 µL/min. Finally, the unreacted active sites on CM5 chip were blocked with ethanolamine (pH = 8.5) for 7 min. The compounds were dissolved and diluted to test concentration (3.1 µM, 6.2 µM, 12.5 µM, 25 µM, 50 µM, and 100 µM) in phosphate buffer (PBS) containing 5% DMSO and then injected into the detection system of Biacore T200 at a speed of 30 µL/min. The association and dissociation time of two ligands with HMGB1 protein was set to be 60 s. The Biacore T200 evaluation software was used to analyze the data, and the 1:1 steady-state affinity model was used to calculate the affinity constants of these two phytochemicals with HMGB1.

### Ultraviolet-visible absorption spectrum

The ultraviolet-visible absorption (UV-Vis) spectra of HMGB1, astragaloside IV, and calycosin were respectively scanned in the wavelength range of 200–800 nm with an optical path of 1 cm. The final concentration of HMGB1 was 3.0 µM, and that of the two main components in *A. membranaceus* was 20.0 µM. The UV-Vis absorption spectrum of PBS (pH = 7.4) was recorded as a reference solution to deduct the influence of the UV absorption of the buffer solution.

### Steady-state, synchronous, and three-dimensional fluorescence measurements

By using fluorescence spectroscopy, the intermolecular interaction and their effect on protein’s spatial conformation could be detected through the changes of excitation and emission spectra and fluorescence intensity of proteins with or without ligand [18]. In this work, astragaloside IV-HMGB1 and calycosin-HMGB1 complex with different ligand concentrations were prepared, and the protein solution without small molecules was used as the control group. The final concentration of HMGB1 was fixed at 3.0 µM, and that of astragaloside IV and calycosin was 2.5–100 µM. The steady-state fluorescence spectra in the 290–400 nm wavelength range were recorded with an excitation wavelength of 273 nm and an optical path of 0.1 cm. And the slit width of excitation and emission was set to 10 nm, and the scanning speed was 1200 nm/min. Each concentration was prepared and measured in triplicate.

In the synchronous fluorescence spectra of tyrosine (Tyr) and tryptophan (Trp), the difference in excitation and emission wavelengths (Δλ = λem -λex) was set to 15 and 60 nm, respectively. At the temperature of 298 K, the Tyr and Trp spectrum of HMGB1 were detected in the wavelength range of 250–350 nm and 240–350 nm, respectively. The three-dimensional (3D) fluorescence spectrum at 298 K was scanned with an optical path of 0.1 cm. The final concentration of HMGB1 for synchronous and 3D fluorescence was 3.0 µM, and the final concentrations of astragaloside IV and calycosin were both 20.0 µM.

### Circular dichroism (CD) spectrum

CD is an important technique to characterize the secondary structure of protein [19]. Usually, the α-helix of protein has two negative shoulder peaks around 208 and 222 nm. According to the change in the CD spectrum, the influence of small molecule ligands on the secondary structure content of HMGB1 can be explored. JASCO-810 circular dichroism spectrometer (JASCO, Tokyo) was used to detect the CD spectrum of HMGB1 before and after the interaction of astragaloside IV and calycosin with HMGB1 in the range of 195–260 nm at 298 K, respectively. The optical path was 1.0 mm and the scanning speed was 50 nm/min. The concentration of HMGB1 was 3.0 µM, and that of astragaloside IV and calycosin were 20.0 µM.

### Molecular docking

ChembioDraw 2014 software was used to draw the chemical structures of astragaloside IV and calycosin. The planar structures were converted to 3D structures by Chem3D, and then MM2 method was used for energy optimization and saved as Mol2 files. HMGB1 structure was retrieved from the Protein Data Bank (https://www.rcsb.org/) (PDB ID: 2YRQ) [20]. The above structures were imported to Maestro 11.8 module of Schrödinger software (LLC, New York, USA). Then two ligands were processed by the LigPrep module to produce different protonation states and stereoisomers [21]. The Protein Preparation Wizard module was used to optimize the target protein HMGB1 to obtain the initial conformation before docking. The Receptor Grid Generation module was used to set the binding sites on the protein surface and generate Grid files according to literature reports [22]. Others were the default parameters of Schrödinger software, and the docking of astragaloside IV and calycosin with HMGB1 was performed sequentially in the Glide XP docking module. The conformational analysis with the highest scoring function was selected based on docking score and visual inspection. Binding free energy is subsequently calculated by Prime MM/GBSA.

### Statistical analysis

Data were expressed as mean SD of at least 3 independent experiments (n = 3). One-way ANOVA was used for comparison among all different groups. When the ANOVA was significant, posthoc testing of differences between groups was performed using Tukey’s test. A *P* value < 0.05 was considered statistically significant.

## Results and discussion

### Inhibition of astragaloside IV and calycosin on HMGB1-induced NO release

**Fig. 1:**
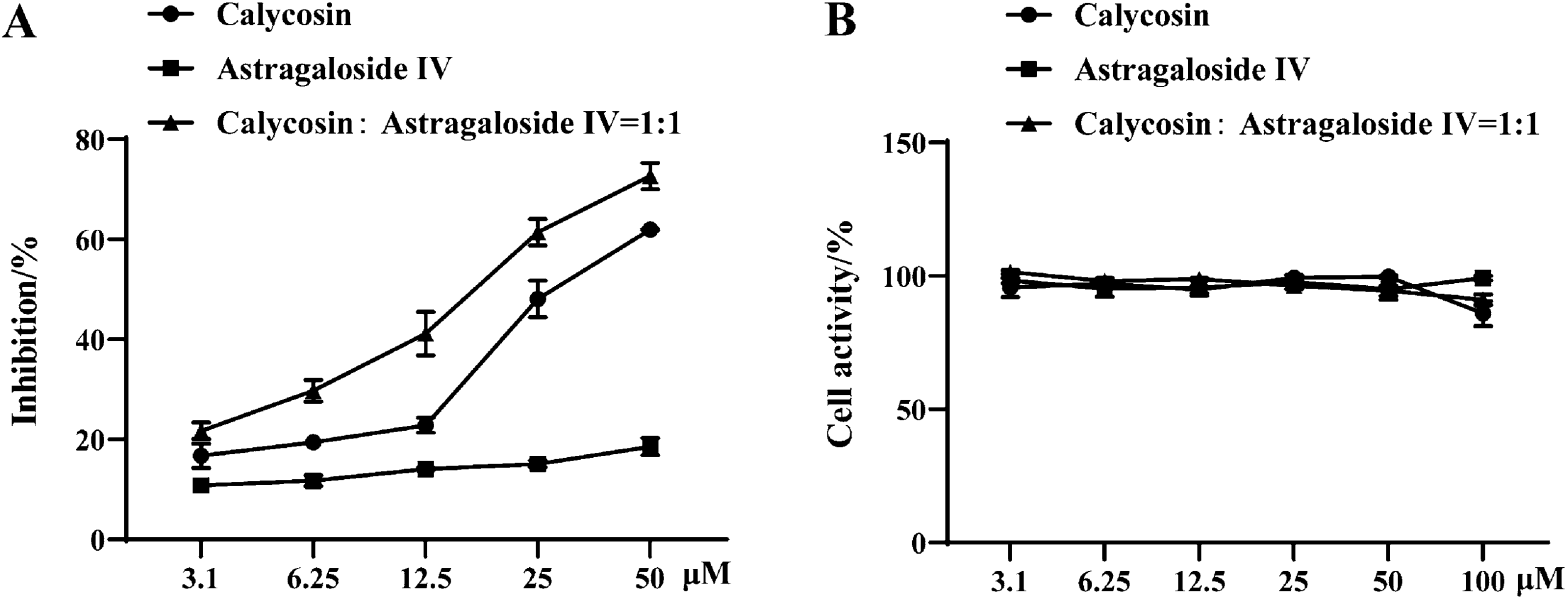
**A** Inhibitory effect of astragaloside IV and calycosin on HMGB1-induced NO release, **B** Cell viability of astragaloside IV and calycosin on RAW 264.7 cells

The long-term and severe inflammatory response is involved in the pathological process of sepsis, arthritis, septic shock, cancer and cardiovascular diseases [23]. As a typical DAMP molecule, extracellular HMGB1 can induce the release of various endogenous cytokines, such as NO, TNF-α, IL-1β, IL-6, etc., thus aggravating the inflammatory diseases [24]. Herein, the inhibition of astragaloside IV and calycosin were evaluated on HMGB1-stimulated nitric oxide (NO) release in RAW 264.7 cells. The results showed calycosin effectively downregulated proinflammatory functions of HMGB1 with an IC_50_ value of 31.79 ± 0.41 µM, whereas astragaloside IV exhibited less potency in HMGB1-induced nitrite release (Fig. 1). To further explore the synergistic effect between two ingredients, astragaloside IV and calycosin were preincubated with HMGB1. As expected, the NO level increase induced by HMGB1 was strongly inhibited with IC_50_ decreasing to 16.89 ± 1.96 µM, indicating that the two main components of *A. membranaceus* had the cooperative therapeutic action on HMGB1-dependent inflammatory response. None of the compounds showed any toxicity and significantly affected NO production without HMGB1 in RAW 264.7.

### SPR direct-binding experiments

**Fig. 2:**
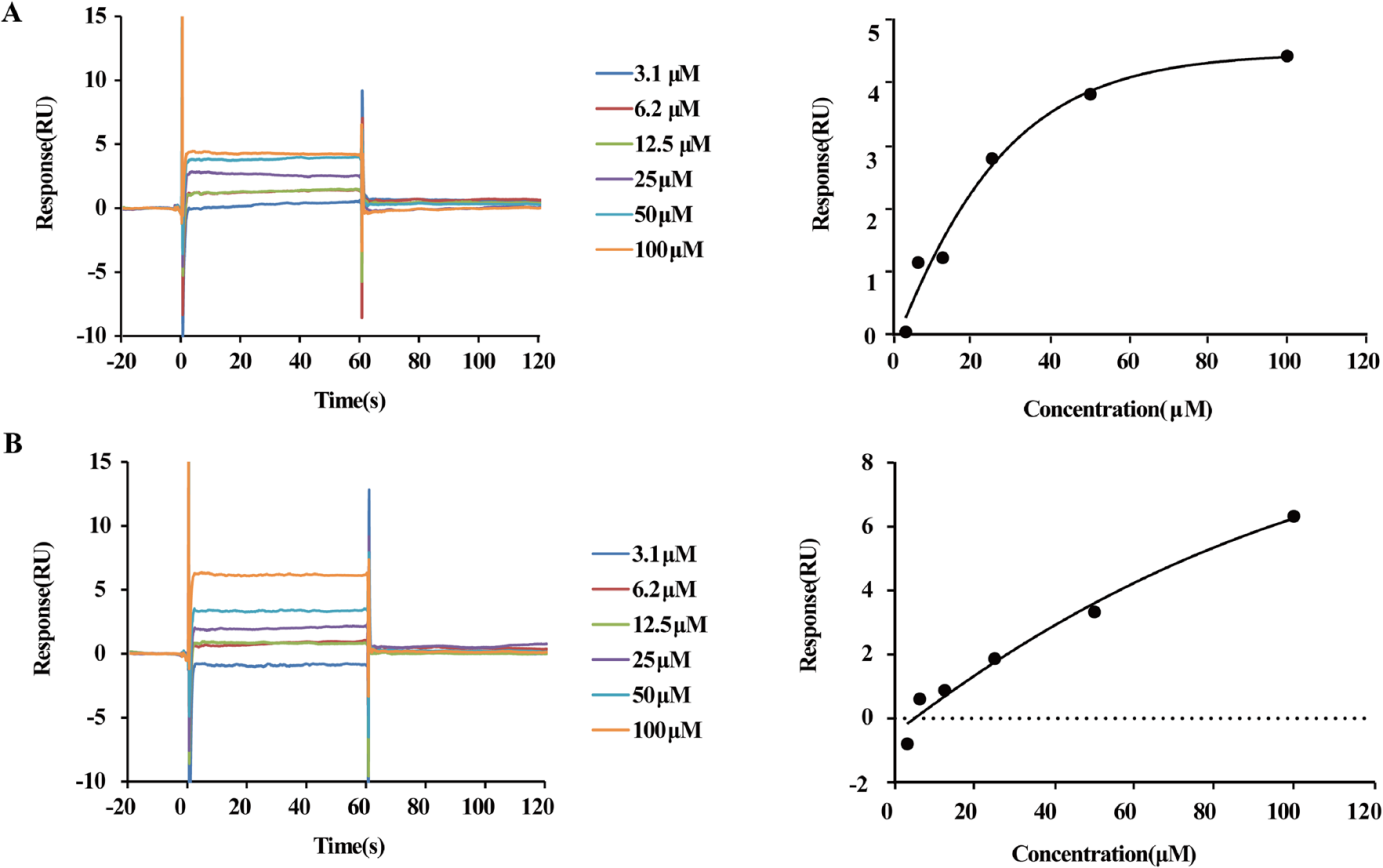
The association-dissociation curve (left) and steady-state fitting curve (right) of Astragaloside IV (**A**) and calycosin (**B**) binding to HMGB1.

To determine whether the inhibition of astragaloside IV and calycosin on HMGB1-dependent inflammation was correlated with their binding ability to HMGB1, the specific affinity between two ligands and HMGB1 was investigated by SPR assay. As shown in Fig. 2, the real-time association and dissociation curves suggested that astragaloside IV and calycosin could interact with HMGB1 at a rapid association and dissociation rate. The dissociation constants (*K*_D_) were calculated as 2.77 × 10^− 5^ M, 1.19 × 10^− 4^ M by steady-state fitting, respectively (Table 1). It can be seen that the affinity between calycosin and HMGB1 is weaker than that of astragaloside IV, that is, a higher concentration of calycosin is needed to saturate HMGB1. Besides, there may be a transient and weak interaction between the two ligands and target protein HMGB1, which was in accord with the mode of action of TCM. Next, the interaction interface and detailed binding mechanism of astragaloside IV and calycosin with HMGB1 will be explored.

**Table 1:** Binding parameters of astragaloside IV and calycosin with HMGB1

Compounds	*K*_D _(M)	Rmax (RU)	offset (RU)	Chi (RU[2])
Astragaloside IV	2.767 × 10^− 5^	5.592	−0.00481	0.125
Calycosin	1.119 × 10^− 4^	13.95	−0.5784	0.362

### Fluorescence spectroscopy

**Fig. 3:**
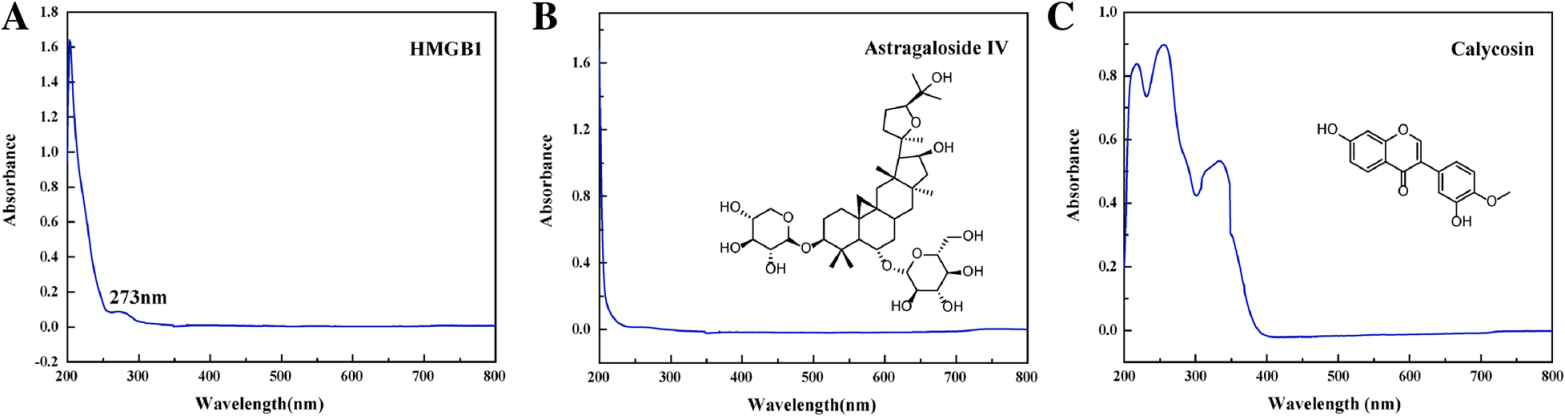
Uv-vis absorption spectrum of HMGB1 (**A**), astragaloside IV (**B**) and calycosin (**C**)

The spectroscopy technology is a powerful tool for the study of drug binding with proteins under physiological conditions with nonintrusive measurements [25]. In this work, the ultraviolet-visible (UV-vis) spectrophotometer was first used to record the maximum absorption wavelength of HMGB1, astragaloside IV and calycosin, respectively. As shown in Fig. 3, two absorption peaks, near 210 and 273 nm were observed for HMGB1 protein. Among them, 210 nm of absorption peak was made up of peptide bond carbonyl happen in n-π* transition in HMGB1 protein. The absorption peak at 273 nm was characteristic of π-π* transition occurring for aromatic amino acid residues. Calycosin presented three obvious absorption peaks around 210 nm, 260 and 360 nm due to its structure containing multiple unsaturated segments. The maximum absorption peak of astragaloside IV was located at 210 nm. 

**Fig. 4:**
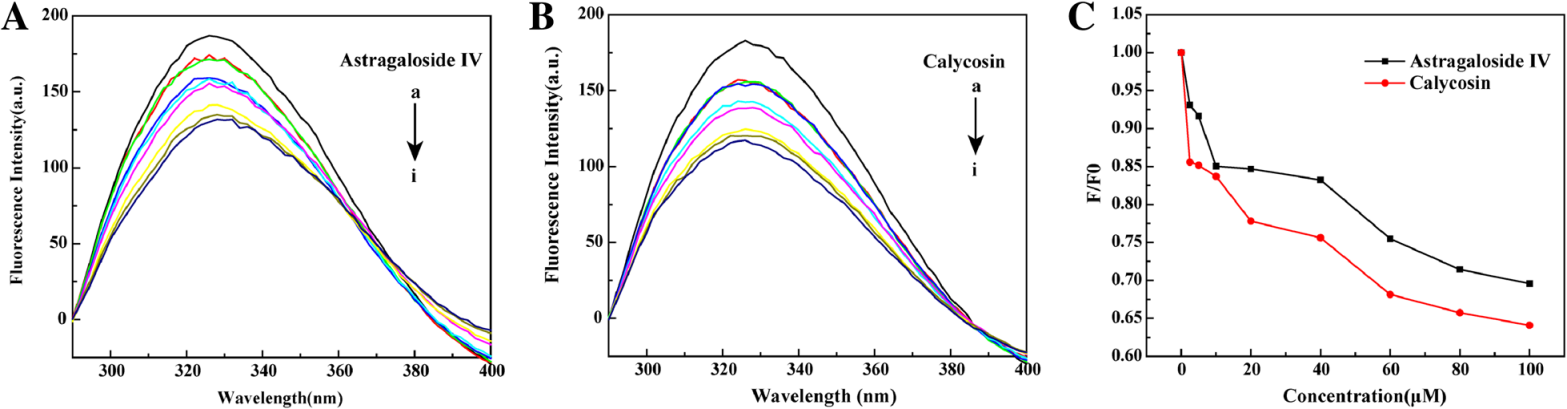
Fluorescence spectrum of HMGB1 in the present of astragaloside IV (**A**) and calycosin (**B**), (**C**) Concentration-dependent quenching on the fluorescence intensity of HMGB1 induced by two ligand molecules. (The concentration of HMGB1 was 3 µM and that of astragaloside IV, calycosin (A-I) were 0, 2.5, 5, 10, 20, 40, 60, 80, 100 µM, λex = 273 nm)

To investigate the influence of the interaction on the HMGB1 conformation and the surrounding amino acid microenvironment, the steady-state fluorescence spectrum was scanned using 273 nm as the excitation wavelength and 290–400 nm as the emission wavelength. The results showed that the maximum emission wavelength of HMGB1 was about 330 nm, indicating that a hydrophobic environment surrounded the Trp residue. Astragaloside IV and calycosin did not emit fluorescence in the wavelength range of 290–400 nm, which did not interfere with the fluorescence spectrum of the HMGB1 protein. In the presence of astragaloside IV or calycosin, the fluorescence intensity of HMGB1 gradually decreased in a concentration-dependent manner, indicating that the two ingredients were bound to HMGB1. Moreover, the addition of astragaloside IV led to a slight red shift of the maximum absorption wavelength of HMGB1 (Fig. 4). A slight blue shift was observed at HMGB1-calycosin complex, indicating that the polarity of the microenvironment around Trp and Tyr residues in HMGB1 decreased after adding calycosin. Based on the above results, it could be speculated that astragaloside IV, calycosin could directly bind with HMGB1 and thus affect the microenvironment of its amino acid residues.

**Fig. 5:**
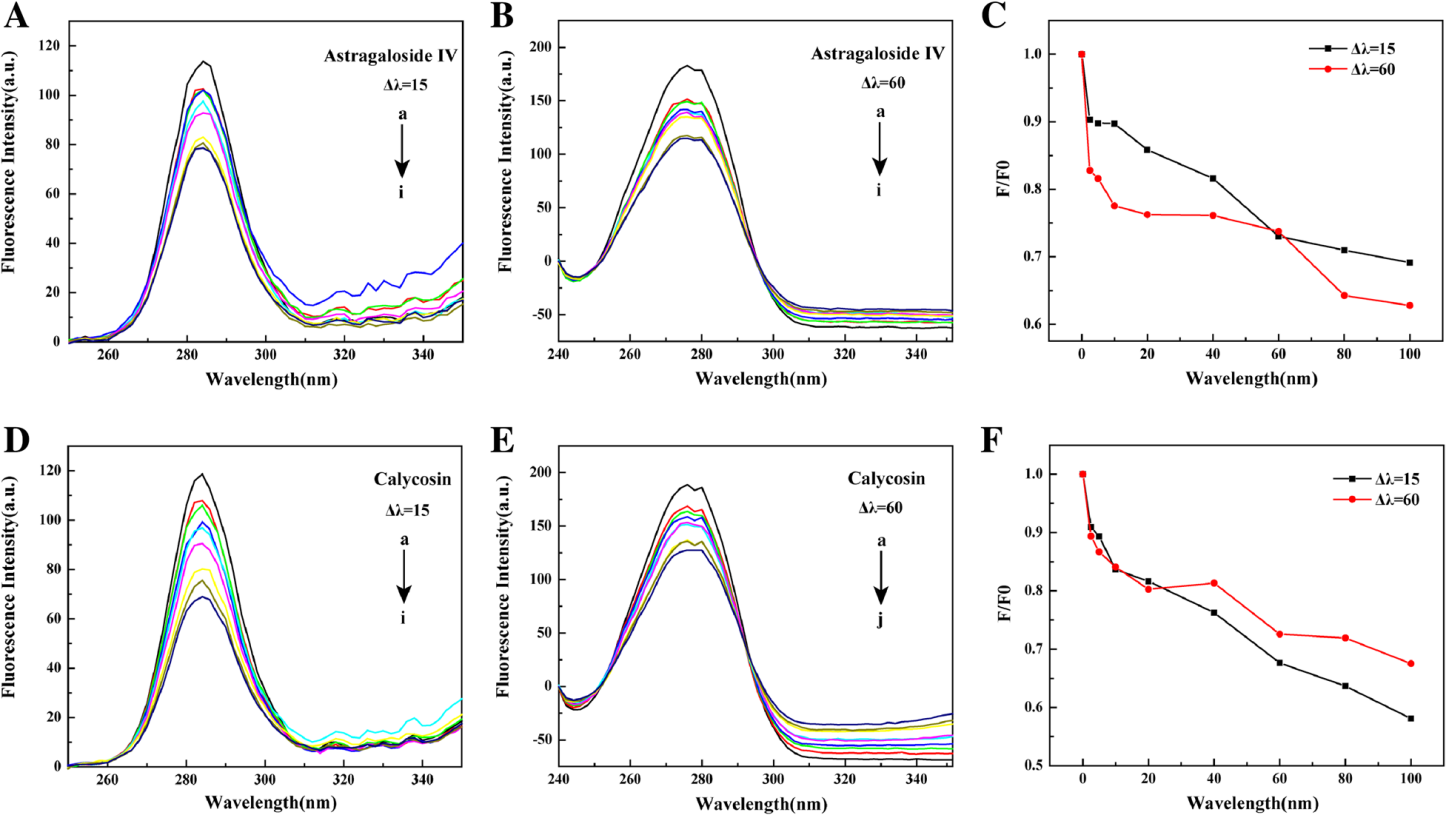
Synchronous fluorescence spectra of astragaloside IV- (**A**-**C**) and calycosin (**D**-**F**)-HMGB1 (Δλ = 15 nm or Δλ = 60 nm) (The concentration of HMGB1 was 3 µM, the concentration of astragaloside IV and calycosin from A-I was 0, 2.5, 5, 10, 20, 40, 60, 80, 100 µM, λex = 273 nm)

In Fig. 5, the synchronous fluorescence spectra of HMGB1 in the presence or absence of astragaloside IV and calycosin were also recorded. When Δλ = 15 nm, it refers to the fluorescence spectrum of Tyr residues; when Δλ = 60 nm, it reflects the fluorescence characteristics of Trp residues. The change in the maximum emission wavelength of HMGB1 protein can reveal the polarity/hydrophobicity around the chromogenic group and HMGB1’s spatial conformation. If the maximum emission wavelength of HMGB1 is red shifted, indicating that the polarity of microenvironment of amino acids is enhanced. In this study, with the increase of concentration of astragaloside IV and calycosin, Tyr and Trp fluorescence intensity of was regular quenching. In detail, astragaloside IV had a more effective fluorescence quenching on Trp residues, while calycosin on Tyr residues microenvironment. However, there was no obvious redshift or blueshift in the maximum emission wavelength, suggesting the binding of two components with HMGB1 had little effect on the polarity and hydrophobicity of the microenvironment.

**Fig. 6:**
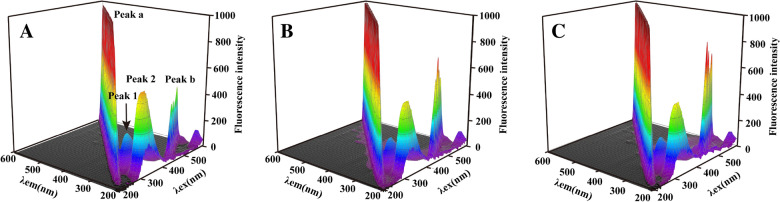
3D fluorescence spectrum of HMGB1 (**A**), HMGB1-astragaloside IV (**B**) and HMGB1-calycosin (**C**) (The concentration of HMGB1 is 3 µM, and that of astragaloside IV and calycosin are 20 µM, 298 K)

The 3D fluorescence spectrum comprehensively presents excitation, emission spectra and fluorescence intensity of information, usually used to explore the effects of small molecules on the spatial conformation of proteins [26]. As shown in Fig. 6, the 3D fluorescence of HMGB1 contains four characteristic peaks. Peak **a** is the Rayleigh scattering peak (λex = λem), and peak **b** refers to the second-order scattering peak (2λex = λem). Peak **1** (λex = 280 nm, λem = 340 nm) reflects the spectral characteristics of Trp and Tyr and the changes in the microenvironment [27]. Peak **2** (λex = 230 nm, λem = 340 nm) derived from the n-π * transition of the polypeptide chain skeleton of the protein, representing the change of the secondary structure of the protein. By observing the changes of four characteristic peaks in the presence of two ligands, the interaction characteristic between them and HMGB1 was studied. In Fig. 6, the fluorescence intensity of peak **1** was reduced by 12.25% and 11.92% (Table 2), respectively, indicating that the addition of astragaloside IV and calycosin impacted the polarity of Trp and Tyr microenvironment. The fluorescence intensity of peak **2** decreased by 13.20% and 13.03%, respectively, suggesting a change in the secondary structure of HMGB1. The results of 3D fluorescence were consistent with the steady-state fluorescence spectrum, which further confirmed that astragaloside IV and calycosin could cause conformational changes of HMGB1 in a direct binding manner.

**Table 2:** Astragaloside IV and calycosin separately and HMGB1 interaction of the 3D fluorescence spectrum data

Fluorescence	HMGB1	HMGB1 + Astragaloside IV	HMGB1 + Calycosin
Peak1	225.4505	197.8366(12.25%)	198.5821(11.92%)
Peak2	485.7708	421.6253(13.20%)	422.4926(13.03%)

### CD spectrum

**Fig. 7:**
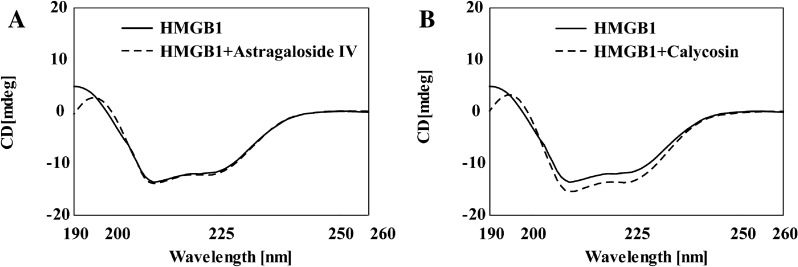
CD spectra of HMGB1, HMGB1-astragaloside IV (A) and HMGB1-calycosin (B)

The interaction between small molecules and proteins likely causes changes in the secondary structure of proteins, which can be detected through CD spectrum. As shown in Fig. 7, HMGB1 protein had two distinct negative peaks at 208 nm (π-π* transition) and 222 nm (n-π* transition), typical of α-helical structures. After adding 20.0 µM calycosin, the CD peak signal of the protein was weakened, indicating a decrease in the α-helix content of HMGB1 protein. It was concluded that calycosin could cause the rearrangement and change of the polypeptide-backbone structure of HMGB1 and thus impair its anti-inflammatory activity. However, astragaloside IV had no significant effect on the CD spectrum of HMGB1, which was consistent with the results of HMGB1 inhibitory activity at the cellular level.

### Molecule docking

**Fig. 8:**
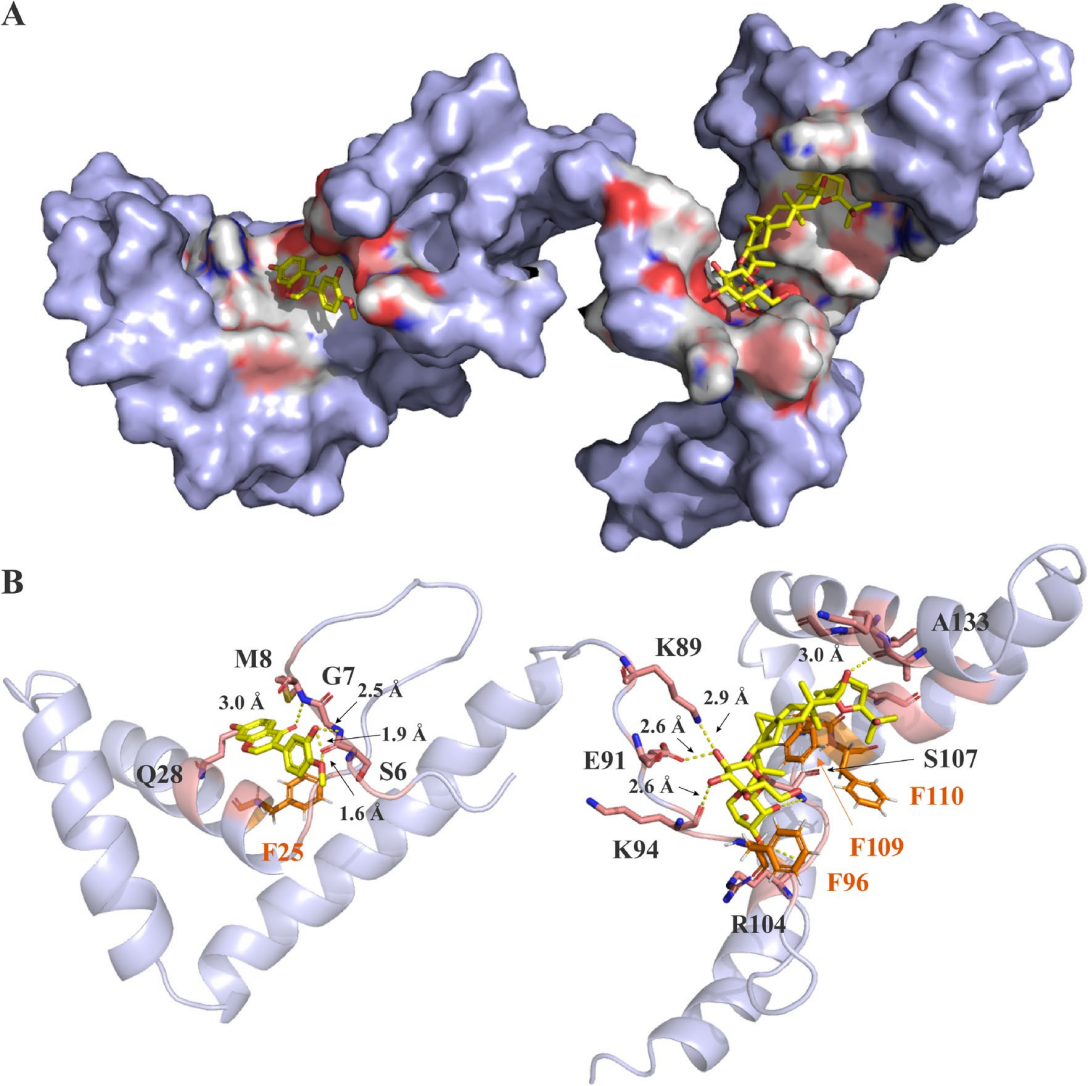
The bind conformation of astragaloside IV and calycosin (yellow) with HMGB1 (key residues are indicated in pink and aromatic amino acids in orange.) (**A**: Surface, **B**: Cartoon)

Numerous studies have demonstrated that herbal extracts as a whole offer better efficacy than equivalent doses of individual active ingredients when used alone, highlighting the significance of synergistic action in herbal therapies. In this paper, to explore how calycosin and astragaloside IV synergetically interact with HMGB1 at the atomic level, semi-flexible docking was performed according to the binding pocket of HMGB1 reported in the literature [22]. The docking results showed that calycosin was mainly bound to the A-box of HMGB1 (Fig. 8A), while astragaloside IV mainly was bound to the B-box domain of HMGB1. The optimal binding conformation of the astragaloside IV-B-box complex has a binding affinity of −7.562 kcal/mol, while that of calycosin-A-box is −7.507 kcal/mol. As shown in Fig. 8B, the C_16_-OH of astragaloside IV interacted with A133 residue of the B-box and formed stable hydrogen bonds with a bond length of 3.0 Å. The OH on the 3-β-D-xylopyranosyloxy formed two additional hydrogen bonds with R104 and S107, while the polar groups on 6-β-D-Glucoside interacted with K89, E91, and K94 (bond lengths of 2.9, 2.6 and 2.6 Å, respectively), which further stabilized the binding of astragaloside IV and HMGB1.

In the HMGB1-calycosin complex, the C_7_-OH forms a stable hydrogen bond with Q28 residue with bond lengths of 2.5 Å, while the hydroxyl on B ring of calycosin showed more favorable interactions with S6 and G7 residues in the HMGB1 binding site by H-bond interactions. In addition, according to the binding conformation, F25 close to calycosin in the A-box domain, and F96, F109, F110 located around the astragaloside IV, are considered as the key residues that lead to fluorescence quenching of HMGB1. The binding free energy of Astragaloside IV/B-box was calculated as -70.060 kcal/mol, and that of calycosin-A-box was −42.938 kcal/mol. These findings reasonably explained that the inhibitory effect of astragaloside IV and calycosin on HMGB1-dependent inflammation after co-incubation was stronger than alone.

**Conclusion**

In this work, the interaction of astragaloside IV and calycosin with HMGB1 protein were studied by multiple spectroscopy and molecular docking. These results showed that both ligands could directly interact with HMGB1 and inhibit its cytokine function cooperatively. Steady-state fluorescence spectroscopy showed that the interaction could dose-dependently quench the endogenous fluorescence of HMGB1. To further explore the effect of two ingredients on the secondary structure of HMGB1, 3D fluorescence and CD spectra of HMGB1 were explored. The results showed that astragaloside IV and calycosin could change the conformation of HMGB1 protein to different degrees, and the effect of calycosin was stronger than that of astragaloside IV. In addition, molecular docking results showed that astragaloside IV and calycosin were respectively bound to different domains of HMGB1. However, astragaloside IV binds more closely with HMGB1 at the atomic and molecular levels, but its cellular activity is lower than that of calycosin. The reason for the above differences may be that the molecular docking ignores the solvent and other influencing factors, or it may be that the regional differences in the interaction modes of two molecules with different structural types, such as whether they interfere with the binding of HMGB1 with the downstream receptors TLR4 or RAGE. For that, molecular dynamics (MD) simulation and animal-level experiments will be executed under the physiological environment in future research.

## Data Availability

All data generated or analyzed during this study are included in this manuscript.
